# Preharvest and Postharvest Applications of Fe-Based Nanomaterials: A Potent Strategy for Improving Pepper Storage

**DOI:** 10.3390/nano15070497

**Published:** 2025-03-26

**Authors:** Zhuang Cheng, Xianzheng Yuan, Xuesong Cao, Zhemin Jia, Fang Hao, Jiayi Chen, Le Yue, Zhenyu Wang

**Affiliations:** 1 Institute of Environmental Processes and Pollution Control, School of Environment and Civil Engineering, Jiangnan University, Wuxi 214122, China; 2 Jiangsu Engineering Laboratory for Biomass Energy and Carbon Reduction Technology, Wuxi 214122, China; 3 Shandong Key Laboratory of Environmental Processes and Health, School of Environmental Science and Engineering, Shandong University, Qingdao 266237, China

**Keywords:** Fe-P nanomaterials, pepper fruit, preharvest, foliar application, storage

## Abstract

Nanomaterials (NMs) hold significant potential for enhancing agricultural production, extending the shelf life, and maintaining the quality of postharvest vegetables and fruits. In this study, after foliar spraying with 1, 10, and 50 mg of L^−1^ Fe-P NMs at different stages (seedling, flowering, and fruit stage), the pepper plant growth was significantly improved. In particular, the foliar application of 10 mg of L^−1^ Fe-P NMs during the flowering stage was found to be an optimal cultivation approach to promote the growth, yield, and freshness of peppers. Compared with the control group, Fe-P NMs increased net photosynthetic rate, plant height, and fruit number by 132.7%, 40.4%, and 265.7%, respectively. The applied Fe-P NMs, at the flowering stage, altered the capsaicin metabolic pathway, upregulating the genes for the synthesis of total phenols, flavonoids, lignans, and capsaicinoids. Consequently, these metabolites, which are beneficial for maintaining the freshness of pepper fruits, were increased. Furthermore, Fe-P NMs at the flowering stage downregulated the abundance of rot-causing microorganisms (*Enterobacter* and *Chryseobacterium*) and upregulated beneficial microorganisms (*Pseudomonas*, *Arthrobacter*, *Sphingobacterium,* and *Paenibacillus*) to change the microbial community structure. This ultimately created a micro-ecological environment conducive to the preservation of pepper fruits. For comparison, during pepper fruit storage, dipping and spraying with Fe-P NM suspensions effectively delayed weight loss and enhanced the growth of beneficial bacteria. Nevertheless, the effect was less pronounced than preharvest foliar application. This study provides insights into the pre- or postharvest application of NMs for improving the preservation performance of pepper fruits.

## 1. Introduction

The global population is estimated to reach 9.6 billion in 30 years. To meet the food needs of this growing population, agricultural production will have to increase by 70% to 100% [1,2]. As the population grows, the demand for vegetables and fruits rises accordingly. Nevertheless, approximately 1.3 billion tons of global food produced for human consumption is wasted each year [3]. Vegetables and fruits account for 33% of this total waste. Moreover, vegetables and fruits are lost at every stage of the food chain. These losses cause an economic cost of 1 trillion USD, an environmental cost of 700 billion USD, and a social cost of 900 billion USD [4]. Besides the economic losses, spoiled produce generates harmful secondary metabolites like mycotoxins and allergens, which are potentially carcinogenic and mutagenic on human health. Therefore, reducing these postharvest losses is crucial for sustainably feeding the world’s population in the future.

Pepper (*Capsicum annuum* L.) is one of the most important vegetable crops in the world. It is not only consumed as a vegetable but also serves as an important condiment. Moreover, it contains a variety of nutrients like carotenoids, proteins, and vitamin C, which has led to a growing demand for pepper consumption. Capsaicin, biosynthesized through the condensation of vanillylamine that originates from the phenylpropanoid pathway and branched-chain fatty acid pathways, is unique to pepper fruits. It contributes to the plant’s resistance to damage caused by mammals and pathogens [5]. Phenylpropane metabolism in plants mainly helps synthesize secondary metabolites including lignin, flavonoids, isoflavonoids, total phenolics, anthocyanin, and plant hormones. The close connection between these compounds and plant health is demonstrated by the improvement in their antioxidant activity and their enhanced ability to defend against pests and diseases [6]. However, pepper loss can occur at all stages from field harvesting to postharvest commercialization. For example, the annual loss of bell peppers is estimated to be 40% [7]. During postharvest storage, it is susceptible to water loss [8], fruit softening [9], pathogenic fungal infections [10], and gas damage [11]. Current fruit and vegetable preservation methods mainly focus on the three following aspects: (1) slowing down the rate of ethylene release and respiration; (2) slowing down water loss and maintaining firmness; (3) increasing antioxidant activity [12]. Nevertheless, traditional preservation technologies have drawbacks such as high investment, operational difficulties, and safety risks. Therefore, it is essential to develop novel preservation technologies to meet the requirements for food safety and freshness [13].

Nanotechnology, emerging as an essential tool in the modern agricultural sector, has been developed for applications in nanopesticides, nanofertilisers, and nanosensors [14]. In recent years, due to a series of unique properties of nanomaterials (NMs), an increasing number of studies have applied nanotechnology to the postharvest preservation of fruits and vegetables [15]. Most of these studies have concentrated on the postharvest treatments of fruits and vegetables to promote preservation capacity by reducing firmness and water loss, controlling respiration rate, and inhibiting microbial growth [16]. A few studies have reported the preharvest application of NMs to promote postharvest freshness, mainly through fruit skin thickening, increasing antioxidant content, and the activation of the salicylic acid (SA) pathway to extend the storage time [17]. Iron (Fe) and phosphorus (P) play crucial roles in plant growth. Compared with their non-nano counterparts, Fe and P composite NMs increase biomass and the tissue Fe and P content in cucumber and maize compared to their non-nano counterparts [18]. Our previous studies have demonstrated that Fe-P NMs can increase fruit quality by improving flavonoid synthesis and accumulation [19]. In this study, the effect of the foliar application of Fe-P NMs on the preservation of pepper fruits was investigated, particularly on the capsaicin synthesis pathway in pepper, including metabolites with antioxidant capacity, phytohormones, and nutrients.

In 2022, the global production of peppers reached approximately 37 million tons, and peppers are widely cultivated in China. The foliar application of NMs has several advantages over traditional methods. For example, it was proven to increase yield and disease resistance while reducing pollution and waste [20]. In this study, we hypothesized that through foliar application, Fe-P NMs could enter the plant and promote photosynthesis, thereby enhancing the yield and quality of peppers. Additionally, by regulating the metabolism and hormone levels of the fruits, Fe-P NMs could prolong the storage period of pepper fruits. The aim of this study was to investigate the impact of Fe-P NMs on the preservation of peppers, including the following aspects: (1) exploring the optimal application system to promote pepper growth, yield, and the fruit storage period; (2) identifying the main mechanisms underlying the promotion of pepper fruit preservation (phenylpropane and capsaicin metabolism, resistance synthesis related genes, and hormone regulation); (3) evaluating the impact of Fe-P NMs on microbial communities in pepper fruits during storage; (4) assessing the effect of dipping or spraying Fe-P NM suspensions on the preservation of pepper fruits as compared to the foliar application. This study not only provides theoretical support for the preharvest application of NMs to enhance fruit preservation but also offers a theoretical basis for the development of fruit and vegetable preservation technologies.

## 2. Materials and Methods

### 2.1. Samples and Reagents

Pepper seeds (Tianwen 3) were obtained from Shandong Fenghong Seed Industry Co., Ltd (Jinan, China). Polyvinylpyrrolidone (PVP, CAS No. 9003-39-8); iron(III) chloride hexahydrate (FeCl_3_·6H_2_O, CAS No. 10025-77-1) and ammonium dihydrogen phosphate (NH_4_H_2_PO_4_, CAS No. 7722-76-1) were purchased from Sinopharm Group Co., Ltd., (Shanghai, China).

### 2.2. Synthesis and Characterization

The NM synthesis was as referred to in our previous study [19], yielding Triiron Tetrairon Phosphate (Fe_7_(PO_4_)_6_, referred to as Fe-P NMs. First, dissolve 0.5 g of PVP, 2.025 g of FeCl_3_·6H_2_O, and 3.45 g of NH_4_H_2_PO_4_ in 10 mL, 20 mL, and 20 mL of deionized water, respectively. Combine the PVP and NH_4_H_2_PO_4_ solutions in a 250-mL round-bottom flask. Then, add the FeCl_3_·6H_2_O solution drop-by-drop from a separating funnel into the mixed solution at a rate of one drop every 6 s, with continuous magnetic stirring. After the addition, stir for another 30 min. Centrifuge and discard the supernatant, transfer the precipitate to a 100-mL polytetrafluoroethylene-lined stainless-steel autoclave, and add 60 mL of ultrapure water. React at 180 °C for 6 h. After cooling, collect the precipitate by centrifuging at 5000 rpm for 5 min and wash it three times with deionized water. Finally, dry the precipitate in a vacuum-drying oven at 60 °C for 2 h to obtain the Fe-P NMs powder (Figure S1). The morphology, the hydrodynamic diameter and Zeta potential, the chemical formula composition, the Fe valence of Fe-P NMs, and the magnetic property were determined by using transmission electron microscopy (TEM, JEM-2100, Nippon electronics Co. Ltd., Tokyo, Japan), dynamic light scattering (Nano-ZS90, Malvern Instruments, Malvern, UK) (Table S1), X-ray diffraction (XRD, Germany Brock AXS Co., Ltd., Karlsruhe, Germany), X-ray photoelectron spectroscopy (XPS, Thermo Fisher ESCALAB 250Xi, Waltham, MA, USA), and VSM analysis (MPMS-3, Quantum Design, San Diego, CA, USA) (Figure S2).

### 2.3. Plant Cultivation, NM Exposure, and Fruit Storage

After germination, the seeds were transferred into pots packed with 2000 g of soil each. The Fe-P NMs suspensions were, respectively, sprayed at the leaves of the 3-week-old (seedling stage), 5-week-old (flowering stage), or 11-week-old (fruit stage) pepper plants with a concentration of 1, 10, and 50 mg L^−1^ after being ultrasonicated for half an hour. Based on the growth index, nutritional index, and water loss rate of pepper fruits during storage (Figure S3), 10 mg of L^−1^ NMs at the flowering stage was further selected as the application method to explore the mechanisms of facilitated pepper fruits keeping fresh. Two controls, including unexposed control and ion control (3.14 mg L^−1^ P_2_O_5_ + 22.3 mg L^−1^ Fe-EDTA, equivalent mass of P and Fe in 10 mg of L^−1^ Fe-P NMs), were set up. Five replicates were used for each treatment. As the plants grew, they were irrigated daily to keep the water abundant. Eventually, we conducted destructive sampling at the ripe stage of the fruit. The leaves, roots, fruits, and postharvest soil were sampled from each plant, immediately frozen in liquid nitrogen, and stored at −80 °C for further analysis.

The harvested fruits with uniform size, color, and weight without mechanical damage were used for the preservation experiment. In addition, pepper fruits with unexposed control were directly treated by dipping or spraying with different concentrations (1, 10, and 50 mg L^−1^) of Fe-P NM suspensions postharvest. All the fruits were stored at room temperature (25 °C). The fruits were taken out and photographed after being preserved for 0, 7, 14, 21, and 28 days, respectively, while their weight loss and firmness were also measured, as described in the Supporting Information (Text S1).

### 2.4. Determination of Plant Growth Parameters

The relative chlorophyll content of the capsicum leaves was recorded according to chlorophyll content (SPAD-502 plus, Konica Minolta Inc., Tokyo, Japan). Photosynthetic parameters including the net photosynthetic rate (*P*_n_), stomatal conductance (*G*_s_), transpiration rate (*E*), and intracellular CO_2_ concentrations (*C*_i_) were measured using a CIRAS-3 portable gas exchange system (CIRAS-3, PP-Systems, Amesbury, MA, USA). After harvest, the number and fresh and dry weight of fruits were measured.

### 2.5. Determination of Total Phenolics and Lignin

The total phenolics of the pepper fruits were extracted and measured using the Folin–Ciocalteu colorimetric method [21]. Briefly, 20 mg of frozen pepper fruits was ground in liquid nitrogen and mixed with 95% methanol before extracting for 48 h in the dark. The mixture was centrifuged at 25 °C 12,000× *g* for 5 min. The supernatant was mixed with 200 μL 10% (vol/vol) Folin–Ciocalteu reagent and vortexed thoroughly. The mixture was then added with Na_2_CO_3_ and incubated at 25 °C for 2 h. The sample was transferred to a clear 96-well microplate for 200 μL and estimated at an absorbance of 765 nm based on the gallic acid concentration. The lignin was extracted and measured according to Fukuda and Komamine [22], as described in the Supporting Information (Text S2).

### 2.6. Quantitative Real-Time PCR (qRT–PCR)

The qRT-PCR was used to analyze the relative expressions of the pivotal genes involved in the phenylpropane pathway (*Pal*, *Ca4H*, *4CL*, *C3H*, *HCT*, *Comt*, *pAmt*), fatty acid pathway (*Kas*, *Acl*, *Fat*, *Acs*), capsaicin synthesis (*AT3*), abscisic acid synthesis (*NCDE1*, *NCDE3*), ethylene synthesis (*ACS1*, *ACO1*), and anthocyanin (*ANT1*, *ANT2*, *AN1*, *TTG1*) in pepper fruits. The sequences of primers were synthesized by Sangon Biotech Co., Ltd. (Shanghai, China) (Table S1). The total RNA of the capsicum fruits was extracted with an RNA extract kit (Takara, Dalian, China) according to the manufacturer’s instructions. Next, the total RNA was reverse-transcribed to cDNA using a cDNA synthesis kit (CW Biotech Co., Ltd. Jiangsu, China). The qPCR was performed on a CFX96 Touch Real-Time PCR System platform (BioRad, USA) with an UltraSYBR Mixture (CWBIO, Beijing, China). The qPCR amplification program was set as 30 s at 95 °C, followed by 40 cycles of 95 °C for 5 s and 60 °C for 30 s. The data were analyzed using the 2^−ΔΔCT^ method.

### 2.7. Element Content, Single Particle Concentration, and Antioxidant Enzyme Activity

The element content was measured by inductively coupled plasma mass spectrometry (ICP-MS, Thermo Fisher, Dreieich, Germany). The oven-dried capsicum fruits were digested in a microwave-accelerated reaction system (MARS 6, CEM, Charlotte, NC, USA) with a mixture of 3 mL HNO_3_ and 3 mL of ultrapure water. The liquid was filtered using a 0.22 μm microporous membrane and diluted to 50 mL for ICP-MS analysis.

The content of Fe-P NMs in plant leaves and capsicum fruits was analyzed using single-particle ICP-MS (SP-ICP-MS, Thermo Fisher, Bremen, Germany). The tissue was placed into 8 mL of 20 mM 2-(N-morpholino) ethanesulfonic acid (MES) buffer with a pH value of 5.0 and then homogenized. Next, 2 mL of macerozyme R-10 enzyme (5%) was added to the homogenate. The resulting mixture was shaken at 37 °C for a duration of 24 h. After that, the supernatant obtained from the mixture was filtered through a 0.45 μm filter membrane. Finally, it was diluted with ultrapure water for SP-ICP-MS analysis (Figure S4).

Superoxide dismutase (SOD), peroxidase (POD), catalase (CAT), and malondialdehyde (MDA) were detected [23], as described in the Supporting Information (Text S3).

### 2.8. Metabolomic Analysis

The metabolites in the fruits were determined by high-performance liquid chromatography–tandem mass spectrometry (HPLC-MS/MS, Thermo Scientific, Dreieich, Germany). The fruit samples were combined with 1.5 mL of a cold extraction solution. This solution consisted of 80% methanol acidified with 0.1% formic acid, along with 0.2 mg/L of 2-chloro-l-phenylalanine. The resulting mixture was subjected to sonication in an ice bath for 30 min. Subsequently, it was centrifuged at 4 °C and 12,000× *g* for 15 min. Following centrifugation, the supernatant was freeze-dried and then re-dissolved in 200 μL of a solvent mixture composed of methanol, acetonitrile, and water in a volume ratio of 4:4:2 (*v*/*v*/*v*). The re-dissolved mixture was vortex-agitated for 1 min. Finally, this mixture was centrifuged again at 12,000 rpm and 4 °C, and the supernatants were carefully collected for further analysis.

### 2.9. Microbial Community Methodology

On the 21st day after the pepper fruits were harvested and stored, the fruits were preserved in liquid nitrogen and then sent to Shenzhen Weikemeng Technology Group Co., Ltd. for sequencing analysis. First, DNA was extracted and detected. Then, the forward primer 799F (AACMGGATTAGATACCCKG) and the reverse primer 1193R (ACGTCATCCCCACCTTCC) were used to perform PCR amplification on the V5-V7 region of the bacterial 16S rRNA. After product purification, library preparation, and library quality inspection, sequencing was carried out on the Illumina platform.

### 2.10. Statistical Analysis

The statistical analysis was conducted using Software 26.0. All experimental results were from at least triplicate biological replicates and presented as mean value and standard deviation. One-way analysis of variance (ANOVA) followed by the Tukey HSD posthoc test (*p* < 0.05) was used to evaluate differences between all treatments.

## 3. Results and Discussion

### 3.1. NM Characterization

The Fe-P NMs exhibited a rod-like structure with a width of approximately 60 nm, which could enter plants and alter the synthesis of secondary metabolites. The hydrodynamic diameter and Zeta potential of the Fe-P NMs were 196.9 ± 55.4 nm and 16.3 ± 0.8 mV, respectively (Figure S2a). X-ray diffraction was used for the phase analysis (Figure S2b). The characteristic peaks were consistent with the annotation information of the characteristic peaks on the standard card of Fe_7_(PO_4_)_6_ [19]. The results of the X-ray photoelectron spectroscopy (Figure S2c) showed that the peaks of Fe 2p_3/2_ and Fe 2p_1/2_ were located at 711.68 eV and 725.28 eV, respectively. Fe^3+^ and Fe^2+^ formed Fe 2p_3/2_ peaks at 713.08 eV and 717.68 eV, respectively. The Fe 2p_1/2_ peak could be deconvoluted into two peaks at 731.38 eV and 726.58 eV, corresponding to Fe^3+^ and Fe^2+^, respectively. The result of the vibrating sample magnetometer (Figure S2d) indicated that the Fe-P NMs were diamagnetic [24].

### 3.2. Effect of Foliar Applied Fe-P NMs on Pepper Growth and Fruit Quality

**Figure 1:**
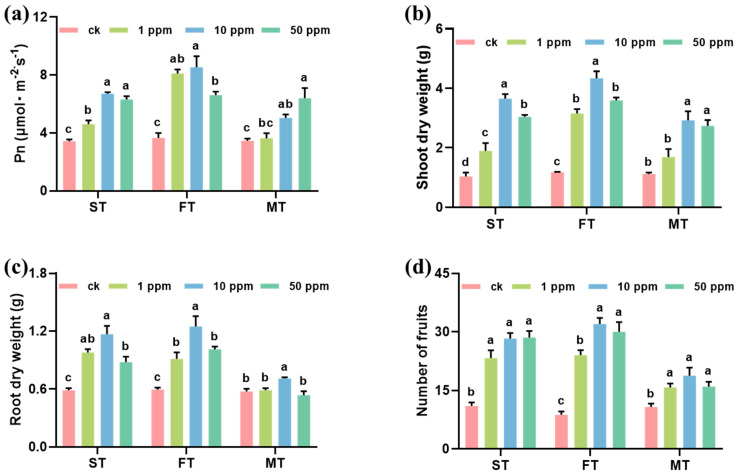
Effects of foliar spray with 1, 10, and 50 mg L^−1^ Fe-P NMs at different stages on the growth indexes of pepper: (**a**) net photosynthetic rate (*P*_n_); (**b**) biomass of shoot; (**c**) biomass of root; (**d**) number of fruits. ST, FT, and MT represent seedling, flowering, and fruiting foliar sprays, respectively. The error line represents the standard error (n = 5), and different letters represent significant differences between different treatments (*p* < 0.05).

After foliar spray with 1, 10, and 50 mg L^−1^ Fe-P NMs at different stages, the pepper plant growth significantly improved. The leave photosynthesis rate (*P*_n_), chlorophyll content, plant height, and fruit number increased by 4.8–132.7%, 3.5–40.2%, 10.4–40.5%, and 46.9–244.4% upon NM exposure as compared with unexposed control, respectively (Figure S3, Figure 1). Among them, 10 mg of L^−1^ Fe-P NMs at the flowering stage had the optimal promotion, significantly increasing *P*_n_ by 132.5%, the chlorophyll content by 41.2%, shoot FW and root FW by 296.7% and 189.9%, shoot DW and root DW by 266.9% and 111.3%, and fruit number by 265.7%, respectively (Figure S3, Figure 1). The results determined by ICP-MS showed that the contents of macronutrients (Na, Mg, P, S, Ca) and trace elements (Mn, Fe, Cu, Zn) showed different degrees of increase under different concentration treatments at different periods. Taken together, the application of 10 mg of L^−1^ Fe-P NMs at the flowering stage showed the most significant effect on the increase in nutrient element contents in pepper fruits, where Na, Mg, P, S, Ca, Mn, Fe, Cu, and Zn contents were elevated by 85.6%, 61.5%, 86.3%, 40.9%, 128.7%, 27.3%, 27.2%, 22.9%, and 35.9%, respectively (Figure S4). In addition, the application of Fe-P NMs at the flowering stage significantly increased the number of particles in pepper leaves, with the number of particles increasing by 21.2%, 48.0%, and 34.5% for the 1, 10, and 50 mg L-1 Fe-P NM treatments, respectively; however, the distribution of single particles in the picked fruits was almost undetectable. This result suggests that pepper fruits grown after the Fe-P NM treatment of leaves were not contaminated with NMs (Figure S4). Further results (Figure S5) showed that 10 mg of L^−1^ Fe-P NM improved the pepper growth better than the ion control containing equivalence Fe and P ion, suggesting the unique “Nano” impact of the promotion. Fe comprises crucial micronutrients, and these have been proven to promote plant photosynthesis and growth [25]. Fe-based NMs have been demonstrated to augment chlorophyll contents and photosynthetic parameters. This enhancement is achieved through multiple mechanisms. Firstly, there is an increase in the utilization and conversion efficiency of energy. Secondly, the electron transfer rate is accelerated. Thirdly, the activity of Rubisco is elevated [26,27]. P plays a crucial role in improving plant reproductive growth, including flower and seed formation [28]. It can also affect the structure and function of the chloroplast membrane, thus improving the nutritional quality of fruit. Quality improvement in pepper fruits can be preventive against pathogens [29]; in particular, the increase in metabolites with antibacterial properties positively contributes to fruit preservation and shelf life. Our study showed that the foliar application of 10 mg of L^−1^ Fe-P NMs at the flowering stage significantly increased the activities of SOD by 17.4% and 7.2%, POD by 77.6% and 12.1%, and CAT by 77.1% and 19.7% in fruits compared to CK and Ion groups. By analyzing the level of MDA in pepper fruits, the fruits were explored to determine if they were subjected to lipid peroxidation and reactive oxygen species accumulation. The results showed that the level of MDA within the fruits applied with Fe-P NMs was reduced by 19.3% and 8.3% compared to the CK and Ion groups, respectively (Figure S6).

**Figure 2:**
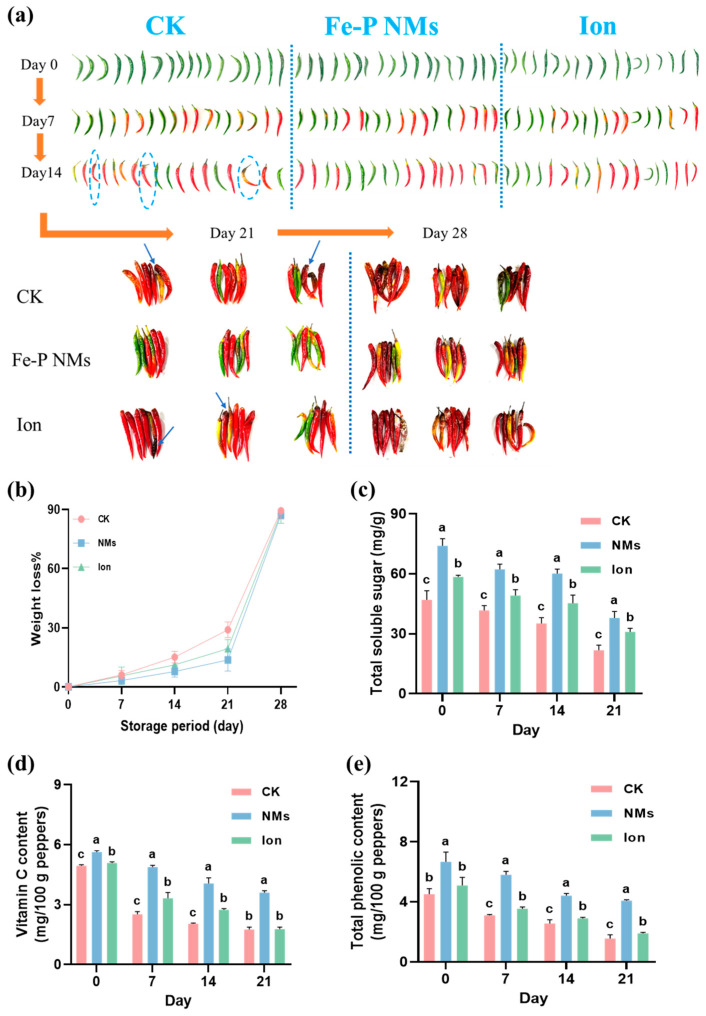
Foliar application of 10 mg of L^−1^ Fe-P NMs at the flowering stage—delayed fruit weight loss and quality deterioration during the storage period of pepper fruits: (**a**) pepper fruit appearance; (**b**) weight loss rate; (**c**) total soluble sugar; (**d**) vitamin C content; (**e**) total phenolic content. The blue dashed circle and arrows refer to the place where the pepper is moldy. The error line represents the standard error (n = 5), and different letters represent significant differences between different treatments (*p* < 0.05).

The appearance of pepper fruits with 10 mg of L^−1^ Fe-P NMs and Ion preharvest application was observed during the storage period. After harvest and storage at 25 °C for 21 days, the pepper fruits in unexposed control started to decay with some brown spots and mold on the exterior, while the Fe-P NM-treated pepper fruits looked smooth, bright, and plump (Figure 2a). However, the ion exposure did not prevent pepper fruits from decay damage. After storage for 28 days, the unexposed pepper fruits extensively decayed with a large area of brown spots and mold, while Fe-P NM0-treated pepper fruits showed less ripening and a better appearance without mold (Figure 2a). After 28 days of storage, the unexposed pepper fruits exhibited the highest percentage of weight loss. This value was 13.7% and 18.7% higher than that of those receiving Fe-P NMs and Ion treatment, respectively (Figure 2b). The content of total soluble sugar, Vc, and total phenolic, which are the indicators of nutrient preservation, were further examined. As shown in Figure 2c–e, these contents decreased steadily in fresh peppers under all treatments as the storage time increased. Specifically, the total soluble sugar content of Fe-P NMs and Ion treatment in fresh-picked pepper fruits was higher than those in the unexposed group by 57.5% and 24.5%, respectively. After storage for 21 days, the total soluble sugar content of the unexposed control, Fe-P NM, and Ion treatments was decreased by 53.7%, 48.8%, and 47.2% as compared to the time they were freshly picked, respectively. Moreover, the total soluble sugar content in those receiving Fe-P NM and Ion treatment was higher than that in the unexposed group by 74.4% and 41.9%, respectively. Similarly, The Vc content of freshly picked pepper fruits that received Fe-P NM and Ion treatments were 13.8% and 2.8% higher than the unexposed group, respectively. After 21 days, the Vc content of the unexposed control, Fe-P NM, and Ion treatments were decreased by 64.4%, 35.9%, and 65.2%, respectively. At this time, the Vc of the Fe-P NM treatment was 105.0% more than the unexposed group, while the Ion treatment showed no significant differences with the unexposed group. A similar trend was also observed in the total phenolic content. After storage for 21 days, the total phenolic content of the Fe-P NM and Ion treatments was higher than that in the unexposed group by 161.7% and 22.0%, respectively.

In summary, the foliar application of 10 mg of L^−1^ Fe-P NMs at the flowering stage significantly enhanced photosynthesis, thereby increasing the biomass and yield of pepper fruits. In addition, the NM treatment reduced the weight loss rate of pepper fruits during storage, delayed the deterioration of fruit quality, and effectively extended the storage period of fruits.

### 3.3. Preharvest Application of Fe-P NMs Improves Capsaicin Synthesis Pathway and Levels of Plant Hormone in Pepper Fruits

**Figure 3:**
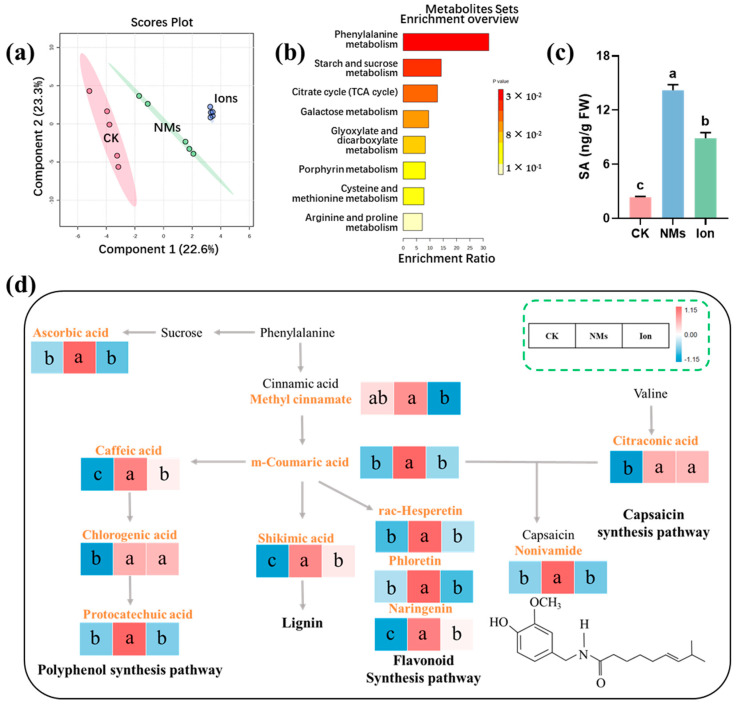
Effect of foliar application on 10 mg of L^−1^ Fe-P NMs at the flowering stage on metabolic pathways in pepper fruits: (**a**) principal components analysis (PCA) of metabolites; (**b**) enrichment analysis of pepper fruits metabolic pathways; (**c**) the content of SA; (**d**) metabolic pathways of lignin, flavonoids, total phenols, and capsaicin synthesis. The error line represents the standard error (n = 5), and different letters represent significant differences between different treatments (*p* < 0.05).

The significantly higher fruit quality maintained in the Fe-P NM treatments is more likely due to the enhanced phenylpropane pathways, which increase the production of secondary metabolites (e.g., total phenolics and flavonoids) in pepper fruits. Principal-coordinate analysis (PCA) revealed a significant difference in the metabolic levels of pepper among the CK, NMs, and ion treatments, accounting for 22.6% of the total variance (Figure 3a). The abundance of 29 metabolites was changed (*p* < 0.05). The abundance of 7 metabolites was significantly decreased, and 22 metabolites were significantly increased upon Fe-P NM treatment at the flowering stage as compared with the unexposed group. Foliar application of 10 mg of L^−1^ Fe-P NMs at the flowering stage mainly improved the processes of the phenylpropane metabolism, the starch and sucrose metabolism, the tricarboxylic acid (TCA) cycle, and the metabolism of amino acids, such as arginine and proline, cysteine, and methionine in pepper fruits (Figure 3b). In the polyphenol synthesis pathway, caffeic acid, chlorogenic acid, and protocatechuic acid contents were increased by 34.3%, 97.3%, and 65.6% with Fe-P NM treatment compared with the unexposed control, respectively (Figure 3d). The accumulation of phenolic compounds around the infected tissues of plants can effectively inhibit the expansion of pathogenic bacteria [30]. In addition, shikimic acid, the primary precursor for the biosynthesis of lignin, was increased by 36.8% compared with the control (Figure 3d). Lignin, a phenylpropane polymer, is the main structural component of secondary vascular tissues and fibers in higher plants. It works with cellulose to strengthen cell walls and improves the resistance of fungi to penetrate plant cell walls [31]. Upon Fe-P NMs exposure at the flowering stage, vast amounts of several metabolites in the flavonoid synthesis pathway were accumulated in pepper fruits after being freshly picked, such as rac-Hesperetin, phloretin, and naringenin, at 36.8%, 22.7%, and 288.6% more than those in the unexposed control, respectively (Figure 3d). Flavonoids have important health-promoting effects by acting as free radical scavengers, thereby protecting plants from oxidative damage [32]. The content of capsaicinoids, which are responsible for the pungent sensation, was increased by 65.6% (Figure 3d). In conclusion, foliar application of 10 mg of L^−1^ Fe-P NMs at the flowering stage influenced the phenylpropane and branched-chain fatty acid pathways in pepper fruits. This led to increased flavonoids and capsaicinoids with antimicrobial and antioxidant properties. As a result, it helped maintain the appearance, nutritional qualities, and flavor of pepper fruits during storage, thus prolonging their storage period.

Further analysis of the metabolic results revealed that NM treatment significantly elevated the relative abundance of metabolites upstream of the salicylic acid (SA) synthesis in the phenylpropane metabolic pathway (cinnamic acid and coumaric acid) (Figure 3a), which was in line with the trend of changes in SA content in fruits, which was upregulated by 5.0 times in the NM treatment group compared with that in the CK group (Figure 3c). SA is an important signal of systemic resistance in plants [33]. As a natural and safe phenolic compound, SA shows great potential in reducing postharvest losses in horticultural crops [34]. SA inhibits cell wall and membrane-degrading enzymes like polygalacturonase (PG), lipoxygenase (LOX), cellulase, and pectinomethyl esterase (PME). This inhibition leads to a decrease in the fruit softening rate [35]. In addition, SA reduces the ripening and senescence rate by inhibiting ethylene synthesis.

### 3.4. Molecular Regulation of Fe-P NM Preharvest Application on the Expression of Genes Involved in Fruit Preservation

**Figure 4:**
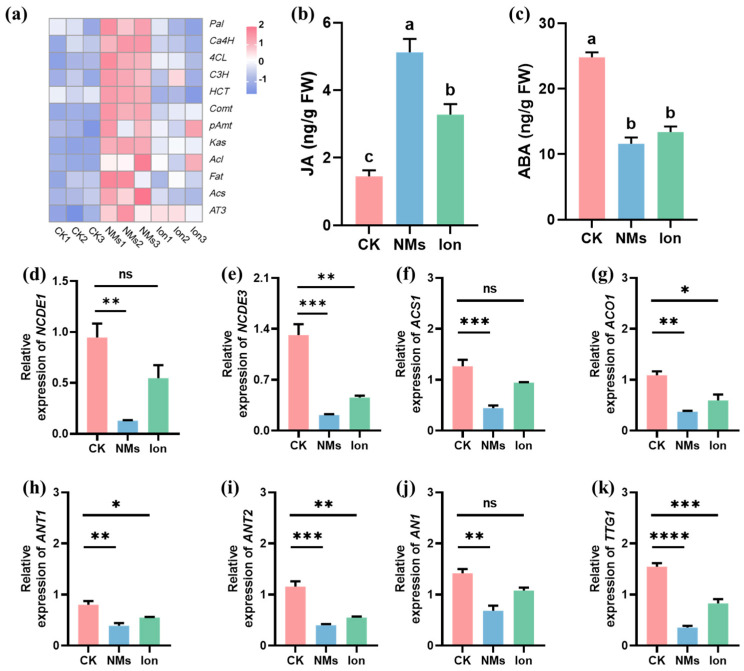
Effects of foliar spray with 10 mg of L^−1^ Fe-P NMs at the flowering stage on pepper fruits: (**a**) the relative expressions of the pivotal genes involved in the capsaicin pathway; (**b**) the concentration of JA; (**c**) the concentration of ABA; the relative expressions of pivotal genes involved in (**d**,**e**) ABA synthesis (*NCDE1*, *NCDE3*); (**f**,**g**) ethylene synthesis (*ACS1*, *ACO1*) and anthocyanin synthesis (*ANT1*, *ANT2*, *AN1*, *TTG1*). The error line represents the standard error (n = 5), and significant differences between treatments are indicated by different letters in (**b**,**c**) and “*” in (**d**–**k**), * *p* < 0.05, ** *p* < 0.01, *** *p* < 0.001, **** *p* < 0.0001, and ns, non-significant.

So far, about 50 genes that may be involved in capsaicin metabolism have been identified [36]. In the study, the gene expressions in the phenylpropane metabolism pathway (*Pal*, *Ca4H*, *4CL*, *C3H*, *HCT*, *Comt,* and *pAmt*), branched-chain fatty acid metabolism pathway (*Kas*, *Acl*, *Fat,* and *Acs*), and capsaicin biosynthesis (*AT3*) were determined (Figure 4a). The phenylalanine ammonia-lyase (PAL), cinnamic acid 4-hydroxylase (C4H), and 4-coumarate-coenzyme A ligase (4CL) play roles in the first, second, and third steps of the phenylpropanoid pathway [37]. Among them, *Pal*, *4CL*, and *HCT* are the core genes within this pathway. These genes are responsible for regulating the metabolism of phenols and lignin in plants [38]. The expressions of *Pal*, *4CL*, and *HCT* in pepper fruits upon Fe-P NM treatment at the flowering stage were significantly upregulated by 0.8, 1.3, and 0.3-fold, respectively, compared with the CK group. In addition, *Comt* and *pAmt* expressions of vanillylamine synthesis in the phenylpropanoid pathway upon Fe-P NM treatment were significantly upregulated by 1.9 and 0.8-fold compared with the unexposed group (Figure 4a). The expression of key genes (*Kas*, *Acl*, *Fat,* and *Acs*) in the branched-chain fatty acid pathway was upregulated by 1.2, 1.7 0.3, and 0.9-folds, respectively, compared to the CK group, probably because soluble sugar content was increased by photosynthesis [39]. The expression of *AT3*, the last key gene in capsaicin biosynthesis, was upregulated by 3.07 folds in the Fe-P NM treatment as compared to the unexposed group (Figure 4a). Above all, the expressions of *Pal*, *Ca4H*, *4CL*, *C3H*, *HCT*, *Comt*, *pAmt*, *Kas*, *Acl*, *Fat*, *Acs*, and *AT3* genes in the fruit were significantly upregulated, and the synthesis of capsaicin and other secondary metabolites could be stimulated upon Fe-P NM treatment compared with the unexposed group, further improving the flavor and nutritional value of the peppers and extending the shelf life.

To further determine the effect of hormones on pepper fruit preservation, the content of jasmonic acid (JA) and abscisic acid (ABA) was measured in freshly harvested pepper fruit. Compared with the CK group, NM treatment significantly increased the content of JA in fruits by 2.5-fold and significantly reduced the content of ABA in fruits by 1.1-fold (Figure 4b,c). The expressions of the marker genes in pepper fruits were further examined. The relative expressions of the ABA-responsive gene in pepper fruits, *NCDE1* and *NCDE3*, in the Fe-P NM treatment, were 6.4-fold and 5.1-fold lower than that of the unexposed group, respectively (Figure 4d,e). The relative expressions of the key genes related to ethylene biosynthesis in pepper fruits, *ACS1* and *ACO1*, were downregulated by 1.8 and 1.9-fold, respectively, upon Fe-P NM treatment at the flowering stage compared with the unexposed group (Figure 4f,g). Fruit anthocyanin synthesis genes (*ANT1*, *ANT2*, *AN1*, *TTG1*) were lowly expressed by 1.1, 1.9, 1.1, and 4.4-fold, respectively, in the Fe-P NM treatment compared with the unexposed group (Figure 4h–k). It has been well established that ethylene plays a key role in the regulation of ripening [40]. During pepper fruit ripening, the color transitions from green to red, attributed to varying levels of chlorophyll and anthocyanin [41]. In the early maturation stages, the fruit’s green color is primarily associated with its chlorophyll content [42]. When the pepper fruits turn red, a large amount of anthocyanin is accumulated and chlorophyll is degraded [43]. Some studies have suggested that the change in anthocyanin content can be caused by ABA [44,45]. Our results demonstrated that the downregulation of *NCDE1* and *NCDE3* expression inhibited ABA synthesis and finally inhibited the expression of anthocyanin biosynthesis-related genes. In summary, Fe-P NMs upregulated the content of SA by affecting the phenylpropane metabolic pathway in the fruits, and SA suppressed the expression of ethylene synthesis genes, delaying the ripening process of pepper fruits in storage; at the same time, they downregulated the content of ABA in the fruits, which led to downregulation of anthocyanin synthesis genes expression, and slowed down the process of pepper fruits changing from green to red. Fe-P NMs promoted freshness preservation by slowing down the fruit ripening process and color change to prolong the storage period and promote the preservation effect.

### 3.5. Microbial Community on the Pepper Fruits During Storage After Fe-P NM Preharvest Application

**Figure 5:**
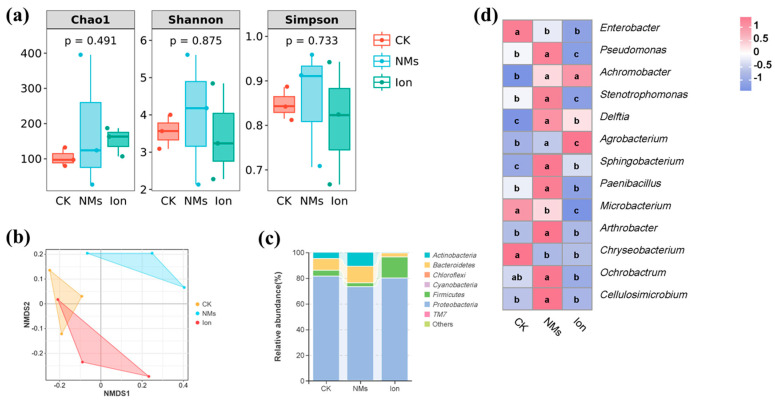
Microbial changes in pepper fruits after foliar spray with 10 mg of L^−1^ Fe-P NMs at the flowering stage: (**a**) Alpha diversity index; (**b**) NMDS analysis; (**c**) relative abundance of dominant bacterial communities at phylum level; (**d**) and relative abundance of dominant bacterial communities in major genera. Different letters represent significant differences between different treatments (*p* < 0.05).

The microbial community composition of pepper fruit might also affect the shelf life of the fruit; thus, the bacterial community of pepper fruits was analyzed. The alpha diversity indices of bacterial communities, including Chao 1 and Shannon and Simpson indices, are shown in Figure 5a. Chao 1 indices and Shannon and Simpson indices reflect species richness and diversity [46]. Compared with the unexposed group, the Chao 1 and Shannon and Simpson indices in the Fe-P NM treatment showed no significant changes. This suggests that, during the storage of pepper fruits, the Fe-P NMs had no impact on the richness and diversity of the bacterial community. Meanwhile, the β-diversity of bacteria was analyzed according to (nonmetric multidimensional scaling) NMDS, and the results indicated that the NM-treated group was completely separated from the other groups; however, the Ion group was not separated from the CK group (Figure 5b). The results showed that the Fe-P NM treatment was able to change the community structure of bacterial microorganisms in pepper fruits compared to the CK group.

For pepper fruits, the relative abundances of the main bacteria are described at phylum and genus levels, respectively. At the phylum level, *Proteobacteria* (78.2%), *Bacteroidetes* (8.3%), *Firmicutes* (8.1%), and *Actinobacteria* (5.3%) were relatively abundant in pepper fruits during storage (Figure 5c). These bacteria were maintained at high levels in apples, peaches, and grapes [47]. *Proteobacteria* was the most abundant of all the treatments, and it served as the dominant phylum within rotten fruits and carried out a diverse range of metabolic activities, including the breakdown of carbohydrates, amino acids, and lipids [46]. Compared with the unexposed groups, the Fe-P NMs group produced a higher abundance of *Bacteroidetes* (43.4%) and *Actinomycetes* (123.3%), which were capable of synthesizing bioactive compounds with antimicrobial activity [48], whereas the abundance of *Proteobacteria*, which are detrimental to fruit freshness, was decreased by 10.4% [49]. At the genus level, the abundance of *Enterobacter* and *Chryseobacterium* in Fe-P NM treatment decreased by 41.5% and 99.7%, respectively, compared to the unexposed group (Figure 5d). Studies have shown that *Enterobacter* [50] and *Chryseobacterium* [51] can cause fruit decay. In contrast, the abundance of *Pseudomonas* and *Arthrobacter* upon Fe-P NM treatment was enhanced by 80.3% and 96.6%, respectively, compared with the unexposed group, and they can be used as biocontrol agents for the effective control of postharvest diseases of fruits [52]. Currently, it is widely acknowledged that the mechanism underlying the antagonistic effects of biocontrol agents against pathogenic bacteria encompasses competition for nutrients and space [53], as well as the production of secondary resistant metabolites [54]. Also, the abundance of *Sphingobacterium* and *Paenibacillus* was upregulated by 97.3% and 150%, respectively, upon Fe-P NM treatment compared with the unexposed group (Figure 5d). *Sphingobacterium* and *Paenibacillus* produce amylase, protease, and chitinase, which attack the fungal cell wall and cause lysis by degrading chitin [55]. In conclusion, a foliar application of 10 mg of L-1 Fe-P NMs at the flowering stage was able to reduce the abundance of rot-prone genera (*Enterobacter* and *Chryseobacterium*) and increase the abundance of beneficial genera (*Pseudomonas*, *Arthrobacter*, *Sphingobacterium*, and *Paenibacillus*) in pepper fruits during storage, and ultimately formed a micro-ecological environment conducive to the preservation of pepper fruits.

### 3.6. Pepper Preservation After Postharvest Application of the Fe-P NM Suspensions

**Figure 6:**
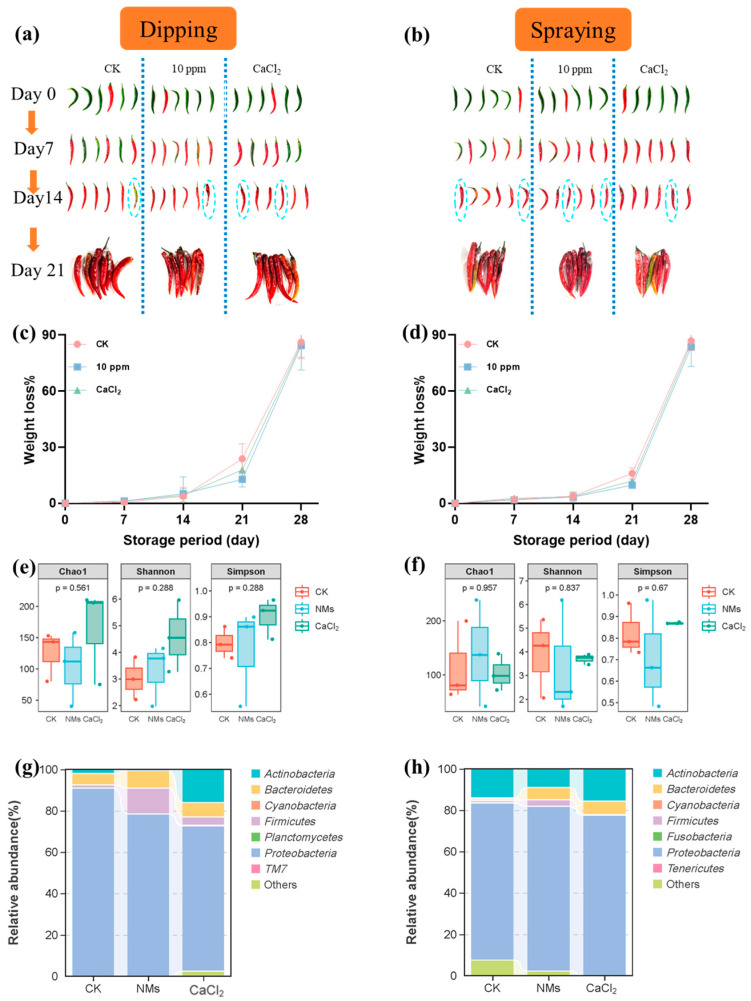
Effects of dipping and spraying with 10 mg of L^−1^ Fe-P NMs on pepper fruit appearance (**a**,**b**), weight loss rate (**c**,**d**), microbial Alpha diversity index (**e**,**f**); relative abundance of dominant bacterial communities at the phylum level (**g**,**h**) during storage. The error line represents the standard error (n = 5).

Two different pepper fruit coating techniques, dipping and spraying (deionized water as a control, 10 mg of L^−1^ Fe-P NMs suspensions, and 2% *w*/*v* CaCl_2_ [56]), were tested in order to choose the most appropriate method to store the pepper fruits. The storage of untreated pepper fruits caused rapid degradation to the fruit’s quality, especially changes in fruit appearance and weight (Figure 6). At 21 days after storage, the treatment with 10 mg of L^−1^ Fe-P NM suspension dipping showed less mold appearance, while the other treatments showed more microbial damage. Weight loss rates of pepper fruits treated with 10 mg of L^−1^ Fe-P NM suspensions, CaCl_2_, and control fruit samples are shown in Figure 6. The results showed that the weight loss rates increased with increased storage time; from day 0 to day 21, the weight loss rates of pepper fruits in the 10 mg of L^−1^ Fe-P NM suspension dipping or spraying group were the lowest. The weight loss rates of the fruit samples exposed to CaCl_2_ were lower than those in the control group during pepper storage (Figure 6).

The microbial communities of pepper fruits after storage upon dipping and spraying were sequenced. The similar tendencies in the preharvest applications observed in the Chao 1 and Shannon and Simpson indices upon dipping and spraying are shown in Figure 6. The results of bacterial β diversity showed significant differences in the bacterial community structure of pepper fruit between Fe-P NM suspension and the control group (Figure S7a,b). Both the dipping and spraying of the Fe-P NM suspensions produced a higher abundance of *Bacteroidetes* (78.4% and 79.6%), *Firmicutes* (12.6% and 3.2%), and *Bacteroidetes* (8.4% and 5.9%) (Figure 6). At the genus level (Figure S7c), after the Fe-P NM suspension dipping, the relative abundance of *Sphingobacterium* and *Paenibacillus* increased by 91.5% and 80%, respectively, which could inhibit fungal growth [53]. Lower relative abundances of *Enterobacter* (98.8%) and *Chryseobacterium* (47.5%) were shown after the Fe-P NM suspension dipping, which caused fruit decay. A similar tendency was observed in Fe-P NM suspension spraying (Figure S7d). There was also a lower relative abundance of *Enterobacter* (73.6%) and *Chryseobacterium* (92.2%), and a higher relative abundance of *Sphingobacterium* (94.1%). Especially, the higher relative abundance of *Streptomyces* (73.5%) was exhibited in sprayed Fe-P NM suspensions compared with the control group. *Streptomyces*, as the microbial antagonistic agent, was reported to produce volatile compounds that effectively control pepper anthracnose under given conditions [57]. In summary, the growth of harmful bacteria in the pepper fruits was inhibited effectively by the dipping and spraying of the Fe-P NM suspensions, and the relative abundance of beneficial bacteria was improved.

## 4. Conclusions

In this study, the foliar application of Fe-P NMs during the flowering stage effectively delayed the weight loss and spoilage of pepper fruits. Multiple mechanisms were included in the processes by which Fe-P NMs affected pepper growth and quality. Firstly, Fe-P NMs promoted photosynthesis in pepper plants, further promoting capsaicin metabolism in pepper fruits. Specifically, the expression of genes involved in the synthesis of resistance substances within the capsaicin metabolism pathway was upregulated. As a result, the contents of lignin, total phenols, flavonoids, and capsaicinoids increased. Meanwhile, plant growth is a complex process, and the promotion of photosynthesis may be interconnected with other physiological regulations. By regulating the contents of phytohormones (SA, JA, and ABA), which are responsible for controlling ethylene and anthocyanin synthesis, Fe-P NMs reduced the synthesis of ethylene and anthocyanin. This led to a delay in fruit ripening and color changes, thereby prolonging the storage period. Additionally, during the storage period, Fe-P NMs altered the abundance of the microbial community in pepper fruits, increasing the presence of beneficial microorganisms such as Pseudomonas, Arthrobacter, Sphingobacterium, and Paenibacillus. These changes ultimately contributed to maintaining the freshness of pepper fruits. Moreover, both Fe-P NM suspension dipping and spraying treatments reduced the weight loss of pepper fruits during storage, delayed pepper fruit spoilage, and downregulated microorganisms capable of causing fruit rot while upregulating beneficial microorganisms. However, the effect was less pronounced than the foliar application during plant growth. This study on the mechanism of NMs in fruit preservation reveals that NMs not only enhance the quality and yield of pepper fruits but also play a crucial role in postharvest preservation. The application of Fe-P NM suspension to pepper fruits provides an environmentally friendly alternative for extending the fruit shelf life.

## Data Availability

The original contributions presented in this study are included in the article/Supplementary Material. Further inquiries can be directed to the corresponding author.

## Supplementary Materials

The following supporting information can be downloaded at: https://www.mdpi.com/article/10.3390/nano15070497/s1, Text S1. Barrier properties, antioxidant properties and antibacterial properties; Text S2. Determination of Weight loss and Fimness; Text S3. Determination of Lignin; Text S4. Determination of Antioxidant Enzyme Activity; Figure S1. The synthesis of Fe-P NMs; Figure S2. Characterizations of the synthesized Fe-P NMs; Figure S3. Growth indexes of pepper; Figure S4. Contents of major elements and trace elements in pepper fruits after treatment with Fe-P NMs; Figure S5. Effects of foliar spray with 10 mg L^−1^ Fe-P NMs and iron-phosphate fertilizer at flowering stage on peppers the net photosynthetic rate P_n_, chlorophyll content, plant height, pepper fruit number; Figure S6. Effects of foliar spray with 10 mg L^−1^ Fe-P NMs and iron-phosphate fertilizer at flowering stage on the activity of antioxidative enzymes; Figure S7. Microbial changes in pepper fruits after dipping and spraying with 10 mg L^−1^ Fe-P NMs suspensions. Table S1. Primer sequences used in this study. (See Refs. [22,23,58,59,60]).
