# Supplementary material for: Preharvest and Postharvest Applications of Fe-Based Nanomaterials: A Potent Strategy for Improving Pepper Storage
Source: Nanomaterials (Basel). 2025 Mar 26;15(7):497. doi: 10.3390/nano15070497 (PMC11990499; doi:10.3390/nano15070497)
Supplement: Supplementary file 1 [file nanomaterials-15-00497-s001.zip › nanomaterials-3521610-supplementary.pdf]

Text S1. Barrier properties, antioxidant properties and antibacterial properties

The test tube containing 5 g of anhydrous calcium chloride, deoxidizer, or potassium hydroxide was sealed with the film samples and the initial weight was recorded. Subsequently, it was stored in a controlled incubator (temperature: 25 °C; relative humidity: 75%) for 48 h, and the weight of the test tube covering films was recorded. The WVP, OP, and CDP were calculated by Eq. (1), Eq. (2), and Eq. (3) [1].

$$\mathrm{WVP} = \frac{\mathrm{W}\times\mathrm{d}}{\mathrm{A}\times\mathrm{t}} \quad (1)$$

$$\mathrm{OP} = \frac{\mathrm{W}\times\mathrm{d}}{\mathrm{A}\times\mathrm{t}} \quad (2)$$

$$\mathrm{CDP} = \frac{\mathrm{W}\times\mathrm{d}}{\mathrm{A}\times\mathrm{t}} \quad (3)$$

Where W represented the increased weight of test tube (g), d represented the thickness of the films (m), A represented the permeation area of the films (m^2), t represented the equilibrium time (s).

The free-radicals scavenging capacities (DPPH) were measured to reflect the antioxidant properties of film samples. 3 mL of film solution was incubated with 3 mL DPPH solution in the dark at 30 °C for 30 min and the absorbance of mixture was measured at 517 nm. The DPPH radical scavenging activity was calculated by Eq. (4) [2]

$$\text{DPPH scavebgingrate}(\%) = \left(1 - \frac{\mathrm{A1} - \mathrm{A2}}{\mathrm{A0}}\right) \times 100 \quad (4)$$

Where A1 is the absorbance of sample with DPPH. A2 is the absorbance of sample with ethanol. A3 is the absorbance of initial DPPH with acetic acid.

The antibacterial properties of film samples were assessed by the plate bacterial killing assays with S. aureus as representative Gram-positive bacteria and E. coli as Gram-negative bacteria. The antibacterial experiment was divided into two experiment groups, where one was performed under UV for 1 h and another was carried out at the darkness for 1 h. The specific experiment process was as below: Firstly, the sterilized centrifuge tube (5 mL), containing 0.5 mL bacterial solution (~10^7 CFU/mL) and the film samples (20 mm × 20 mm), was placed to an incubator at 37 °C with stirring at the speed of 190 rpm under UV or at the darkness. After 1 h, 150 μL of above treated bacterial solution was taken, evenly spread on LB of Petri dish, and then incubated at 37 °C for 16 h to form visible colonies. Moreover, the method of gradient dilution was used to counting the corresponding colonies. The

blank group (CK) was treated with similar steps with experiment group, while without the addition of film samples into bacterial solution. Finally, the relative bacteriostatic rate was calculated by Eq.(5) [3]

$$\text{Relative bacteriostatic rate (\%)} = \frac{(\text{N0} - \text{N1})}{\text{N0}} \times 100 \qquad (5)$$

Where N0 and N1 represented the bacterial colonies number of experiment and blank groups, respectively.

Text S2. Determination of Weight loss and Fimness

The initial weight of fruit was recorded as M0, and its weight after storage was recorded as M1. The weigh loss was calculated by Eq. (6).

$$\text{Weight loss (\%)} = \frac{\text{M0} - \text{M1}}{\text{M0}} \times 100 \qquad (6)$$

The firmness of fruit during storage was measured by using a texture analyzer, where specific parameters were as below: puncture speed: 0.5 mm/s; puncture depth: 7 mm. The firmness of fruit was expressed as N.

Text S3. Determination of Lignin

Lignin was extracted and measured from ground plant tissues by the method of Fukuda and Komamine [4].Specifically, 100 mg of fresh maize leaves were ground to power under liquid nitrogen and transferred to 2 mL centrifuge tubes, which, were added with 1.5 ml 95% ethanol, centrifuged, and the supernatant removed to clean the samples, and repeated three times. After centrifugation, the sediment was collected, air dried, andadded with 0.2 mL 25% acetyl bromide (acetyl bromide: ice acetic acid = 25:75, V/V) solution, placed it in a 70℃ water bath for 30 min, and mixed it upside down every 10 min to ensure that full immersion of the precipitate with the solvent. Thereaction was terminated by adding 0.16 mL 2 M NaOH. Next, 2 mL ice acetic acid and 0.04 mL hydroxylamine hydrochloride (521.175 g/L) were added to the samples, well shaken, and centrifuged at a speed of 1000 rpm for 10 min. Finally, 0.1 mL of the supernatant was taken and diluted by adding 2.0 mL ice acetic acid. The absorbance of mixture was measured at 280 nm.

Text S4. Determination of Antioxidant Enzyme Activity

The antioxidant enzyme activity of fruits was determined according to the method of Luo et al. [5] and slightly modified. Firstly, for the extraction of enzyme

solution, 200 mg of plant samples were homogenized in 1.6 mL of pre-cooled phosphate buffer (pH=7.8), and the supernatant was used as the enzyme solution after vortex at 4 °C and centrifugation at 12000 rpm for 20 min.

For SOD activity, the crude enzyme was mixed with L-methionine, nitroblue tetrazole, riboflavin, and EDTA-Na_2. The mixture was exposed under a fluorescent tube lamp for 20 min, and then determined by a multifunctional microplate at 560 nm. SOD activity takes the amount of enzyme required to inhibit NBT photoreduction by 50% as one unit of SOD.

For POD activity, the crude enzyme was mixed with 200 mM phosphate buffer solution (pH 6.0) containing guaiacol solution and 30% H_2O_2, the mixtures were immediately measured in the wavelength of 470 nm for 2min each 30s by a multifunctional microplate reader. The increase in OD value of 0.01 per minute was considered as one unit of POD.

For CAT activity, the absorbance of 200 μL of the reaction mixture (15 mM phosphate buffer (pH 7.0), 0.05% H_2O_2, and 6.67 μL enzyme extracts) was recorded for 3 min at 240 nm. The reduction of 0.01 of OD value per min was used as one unit of CAT.

Malondialdehyde (MDA) was estimated according to a previous study. 3 Briefly, 100 mg of maize fresh leaf were ground into powder with liquid nitrogen and added 1.5 mL of pre-cooled 0.1% trichloroacetic acid (TCA). After centrifuging at 4 °C, 10,000 rpm for 10min, the supernatant (0.25 mL) reacted with 0.5 mL of 20% TCA and 0.5 mL of 0.5% thiobarbituric acid (TBA) at 95°C for 30 min. Subsequently, the mixture was cooled down on ice before measuring absorbance at 450 nm, 532 nm and 600 nm with a multifunctional microplate reader (Varioskan Lux, Thermo Scientific, Finland). MDA content was determined using the following equation: C_{MDA}(μmol/L)=6.45*(A $bsorbance_{532}$-$Absorbance_{600}$)-0.56*$Absorbance_{450}$.

References

1. Chang, X., et al., Physicochemical and antimicrobial properties of chitosan composite films incorporated with glycerol monolaurate and nano-TiO2. Food Hydrocolloids, 2021. 119: p. 106846.

2. Lin, W., et al., A dual-function chitosan packaging film for simultaneously monitoring and maintaining pork freshness. Food Chemistry, 2022. 392: p. 133242.

3. Xu, H.S., et al., A new nanohybrid particle reinforced multifunctional active packaging film for efficiently preserving postharvest fruit. Food Hydrocolloids, 2023. 144.

4. Fukuda, H. and A. Komamine, Lignin synthesis and its related enzymes as markers of tracheary-element differentiation in single cells isolated from the mesophyll of Zinnia elegans. Planta, 1982. 155: p. 423-430.

5. Luo, X., et al., Nitrogen-doped carbon dots alleviate the damage from tomato bacterial wilt syndrome: systemic acquired resistance activation and reactive oxygen species scavenging. Environmental Science: Nano, 2021. 8(12): p. 3806-3819.

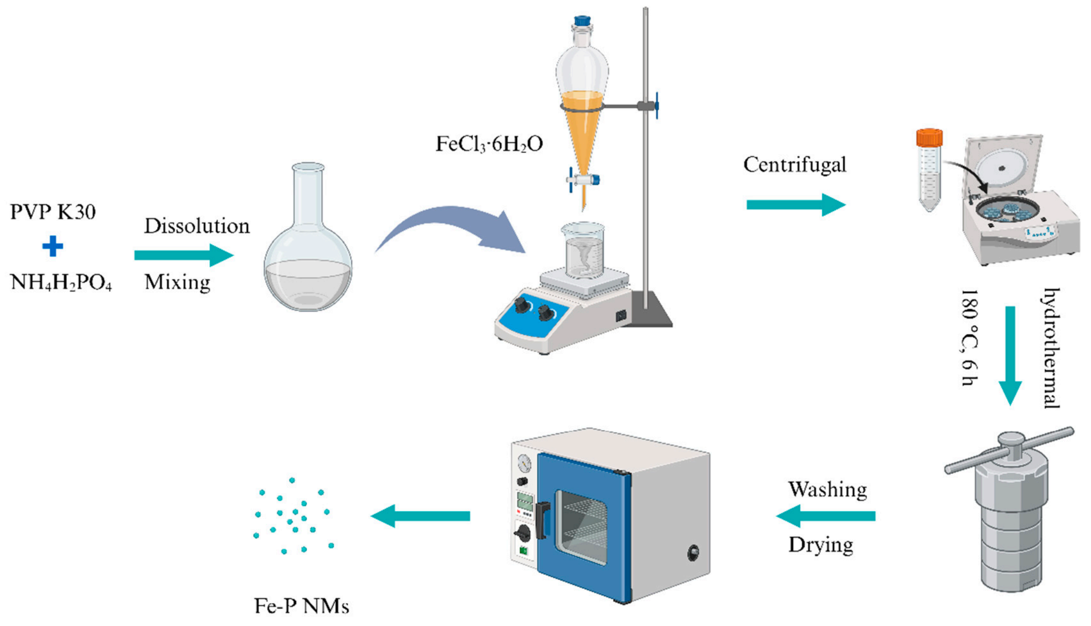

Figure S1. The synthesis of Fe-P NMs

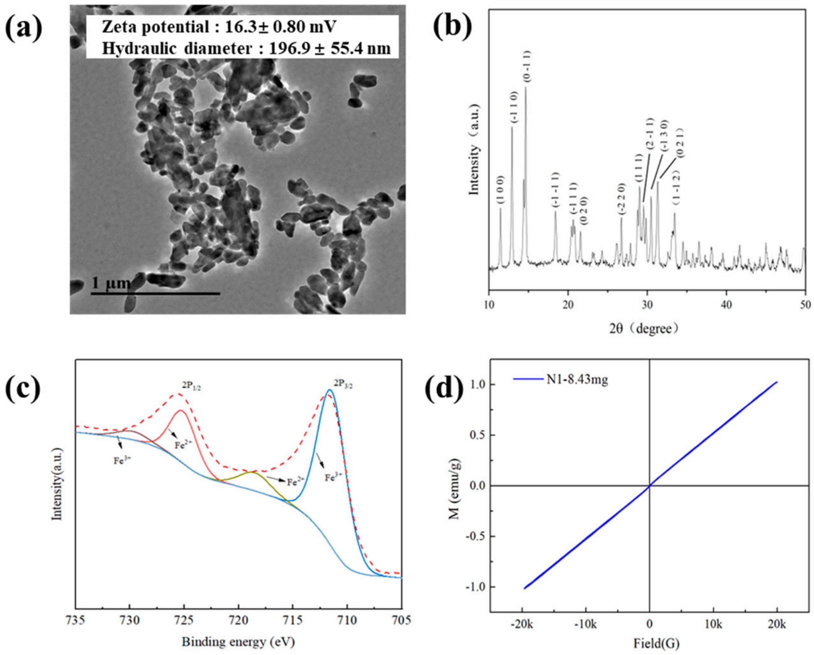

Figure S2. Characterizations of the synthesized Fe-P NMs. (a) TEM image; (b) XRD pattern; (c) XPS diagram of the Fe 2p; (d) VSM image

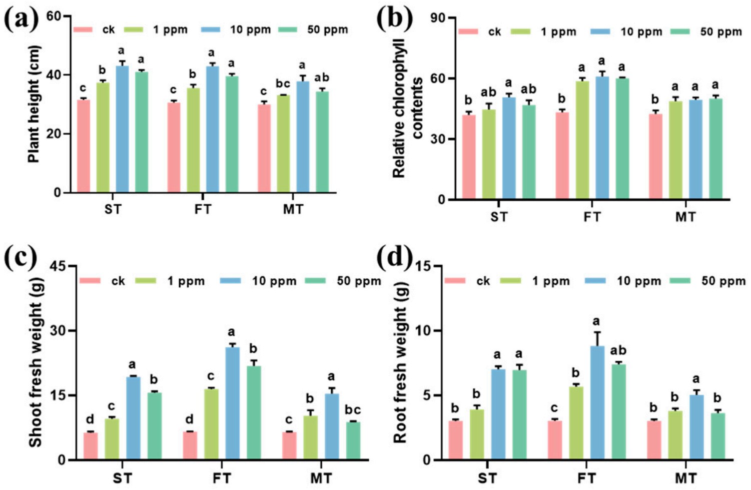

Figure S3. Growth indexes of pepper plants. (a) Plant height; (b) Relative chlorophyll contents; (c) Shoot fresh weight; (d) Root fresh weight. ST, FT and MT represent seedling, flowering and fruiting foliar sprays, respectively. The error line represents the standard error (n = 5), and different letters represent significant differences between different treatments ($p < 0.05$).

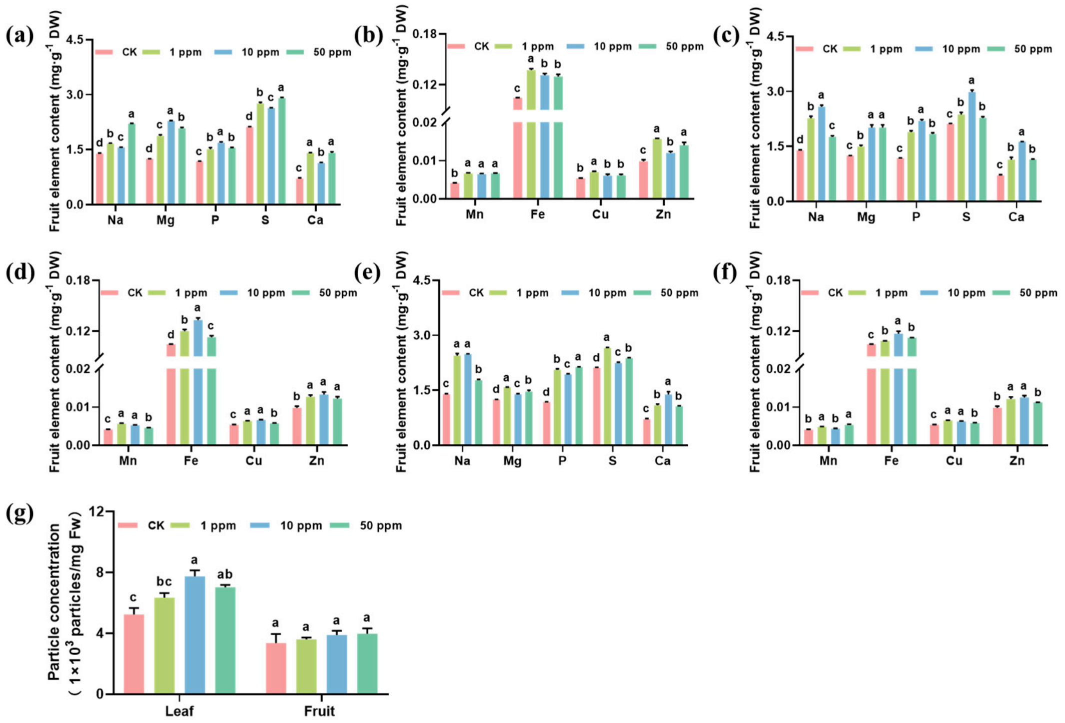

Figure S4 Contents of major elements and trace elements in pepper fruits after treatment with Fe-P NMs at seedling stage (a, b), flowering stage (c, d); and fruit stage (e, f); And number of nanoparticles in pepper leaves and fruits treated with Fe-P NMs at flowering stage (g). The error line represents the standard error (n = 5), and different letters represent significant differences between different treatments ($p < 0.05$).

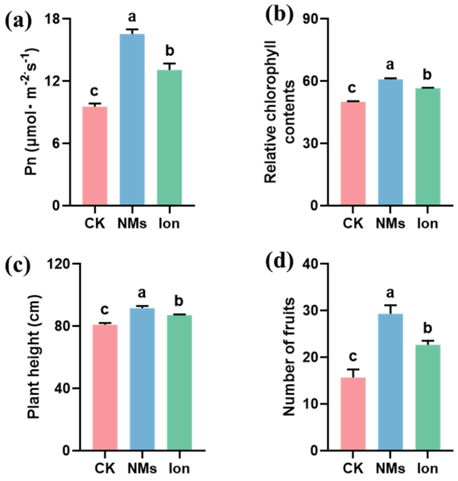

Figure S5. Effects of foliar spray with 10 mg L^{-1} Fe-P NMs and iron-phosphate fertilizer (3.14 mg L^{-1} P_2O_5 and 22.3 mg L^{-1} Fe-EDTA) at flowering stage on the net photosynthetic rate P_n (a), chlorophyll content (b), plant height (c), pepper fruit number (d). The error line represents the standard error (n = 5), and different letters represent significant differences between different treatments ($p < 0.05$).

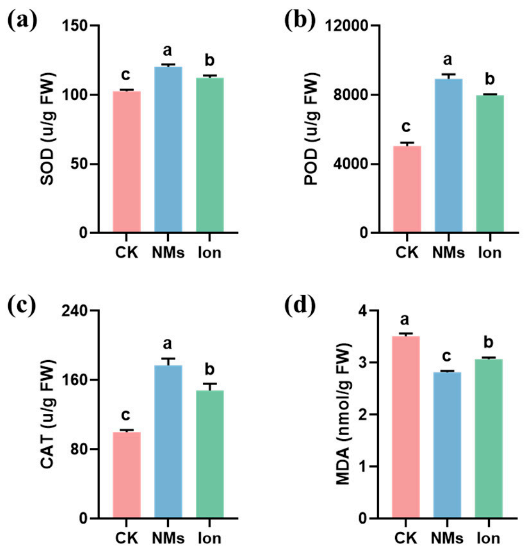

Figure S6 Effects of foliar spray with 10 mg L^{-1} Fe-P NMs and iron-phosphate fertilizer (3.14 mg L^{-1} P_2O_5 and 22.3 mg L^{-1} Fe-EDTA) at flowering stage on the activity of antioxidative enzymes. The content of SOD (a), POD (b), CAT (c), and MDA (d) in pepper fruits. The error line represents the standard error (n = 5), and different letters represent significant differences between different treatments ($p < 0.05$).

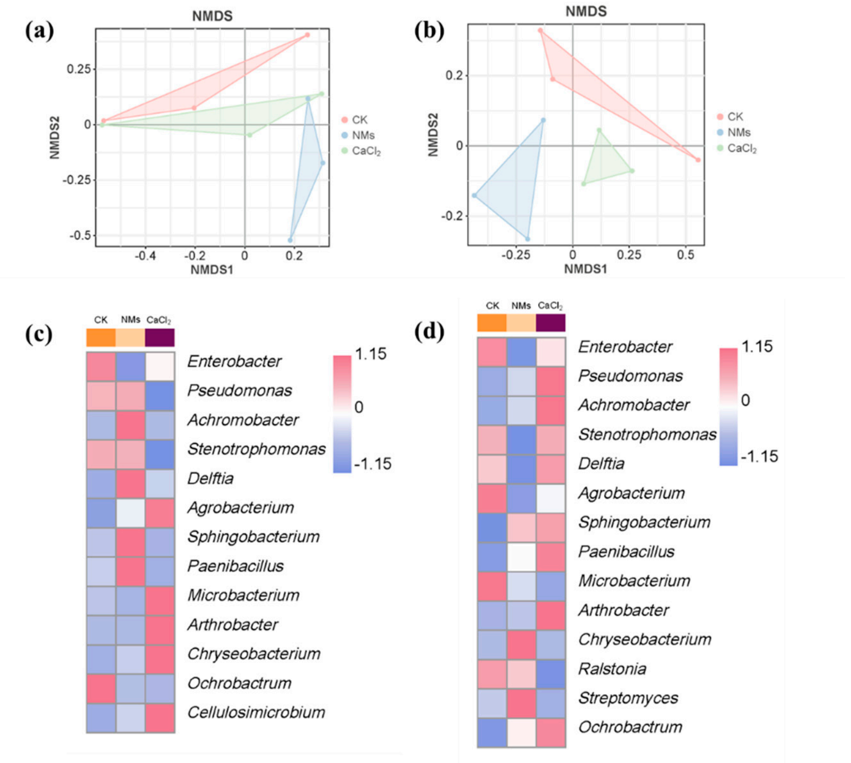

Figure S7 Microbial changes in pepper fruits after dipping and spraying with 10 mg L^{-1} Fe-P NMs suspensions. NMDS analysis upon (a) dipping and (b) spraying treatment; relative abundance of dominant bacterial communities in major genera upon (c) dipping and (d) spraying treatment. Different letters represent significant differences between different treatments ($p < 0.05$).

Table S1. Primer sequences used in this study

Name	Primer sequence (F)	Primer sequence (R)
Actin1	TGTCCATCTGCTCTCTGTTG	CACCCCAAGCACAATAAGAC
Pal	ATTCGCGCTGCAACTAAGAT	CACCGTGTAAGGCCTTGTTT
Ca4H	CTTGGTTAACGCTTGGTGGT	CCGAATGGAAGGAATCTGAA
4CL	GGACCGATTGAAGGAATTGA	GGACAACAGCAGCATCAGAA
C3H	GCCATCTTCTGCACCATTTT	GGCCTGTAATGGAGTCCTCA
HCT	ATGCAGGGATGAAGATGGAC	TAATCAACGGCCGGAATAAG
Comt	CCTGCGAATGGAAAAGTGAT	TCTTTGCCTCCTGGGTTATG
pAmt	TGGCAGAGTGATATGGAACC	GCGCACCATAAACCAGATAA
Kas	GAAAGAATGATCGTGCTTG	GTTGGCCAAGGTTAGCATCT
Acl	TTCCTTCAAGCACAACCAGA	GCGAGTAGCTGGCTTCATTC
Fat	TGATGGATTTGCGACTACCC	CCCAATCCTGCCTTCACTTT
Acs	TGGCTCAGCTGAATTTGTTG	TAACCCGTGAACGTGAAACA
AT3	AGAAGGGAAACTGCCATTTG	TCTTTCAGGTCTTCCCCATC
NCDE1	GAAAAGGAATGGAAATCGGA	CGGGGACGTATATTCTAAAC
NCDE2	AGTACCACCAAAAATTATAGG	TCTGAGCTTTCCGAATC
NCDE3	AAAGAGTGCCATATGGTT	TCAAGAAGGGTATTCAATAGA
ACS1	ACAGCCTCTCTAAGGATCTTGGTCT	TTTGGTTCTCGGCTATGTAGTTCTT
ACO1	GGAGAAGATGACCAAGGAT	GGAAATGTTTGAGGAAGGAA
ANT1	CGTTGCAAGTAAGGAAAGGTGC	CTAGCAGGAACAAGGTGCCA
ANT2	TTGTGAAAAGCCAACAGCCG	CCCAACCATCACTCTGTCCC
AN1	ACCTCCAATCCCACCACCTTT	TTCTCCCGCAATTCTACCATC
TTG1	AGGCATCAAGCGAGTGTGAA	CAAATAAGCGCCTGCCCATC
